# *Osteopathic Medicine and Primary Care *looks forward to 2009

**DOI:** 10.1186/1750-4732-3-2

**Published:** 2009-02-04

**Authors:** John C Licciardone

**Affiliations:** 1 The Osteopathic Research Center, University of North Texas Health Science Center, Fort Worth, TX, USA

## Abstract

*Osteopathic Medicine and Primary Care*, which enters its third year of operation in 2009 under the umbrella of BioMed Central, continues to promote and advance open access publishing through universal online access without charge, indexing in PubMed and archiving in PubMed Central, retention of authors' copyright, and expeditious peer review. Notable accomplishments during 2008 included a median lag time of four months from initial manuscript submission to publication, designation of eight articles as "highly accessed," and achievement of a balanced proportion of publications in our core topic areas of osteopathic medicine and primary care. In October 2008, Springer Science+Business Media, a major publisher of journals in science, technology, and medicine, acquired the BioMed Central Group. Our 2009 Editorial Board is presented herein, as well as a new mechanism for posting book reviews on the *Osteopathic Medicine and Primary Care *website. We continue to encourage manuscript submissions and reader comments on our articles. Waivers or discounts of article processing charges are available via several mechanisms for eligible authors who submit qualified manuscripts.

## *Osteopathic Medicine and Primary Care*

*Osteopathic Medicine and Primary Care *enters its third year of publication with renewed vigor in providing rapid and universal dissemination of peer-reviewed research and scholarly work in primary care, particularly as it impacts osteopathic medicine, via our journal website at . Some of the major reasons for authors to publish in *Osteopathic Medicine and Primary Care *include universal online access without charge, indexing in PubMed and archiving in PubMed Central, retention of copyright, and expeditious peer review.

Our goal in working with the 2009 Editorial Board  is to make *Osteopathic Medicine and Primary Care *the leading journal in its field. We are pleased to report that last year *Osteopathic Medicine and Primary Care *achieved a balanced proportion of publications pertaining either to primary care or uniquely osteopathic topics. Also during 2008, the most viewed article in *Osteopathic Medicine and Primary Care *by Hruby and Hoffman [1] surpassed 5,000 accesses via our journal website. When also considering our mirror site at PubMed Central, 10,000 or more viewers may have already accessed this article. The article presaged the workshop entitled, "Avoiding disaster: osteopathic approach to the flu pandemic," that was recently held in October 2008 in conjunction with the 113^th ^Annual American Osteopathic Association Convention and Scientific Seminar in Las Vegas, Nevada [2]. Thus far, about one-third of all articles published in *Osteopathic Medicine and Primary Care *have earned the "highly accessed" designation independently conferred by BioMed Central [1,3-9].

## The open access publishing environment

*Osteopathic Medicine and Primary Care *is an independent, open access journal published under the umbrella of BioMed Central , the leading global open access publisher. In October 2008, Springer Science+Business Media  acquired the BioMed Central Group. Springer is the second-largest publisher of journals in the science, technology, and medicine (STM) sector. Springer originally launched Springer Open Choice™, its novel open access offering for authors and institutions, in 2004. Its acquisition of the BioMed Central Group signals that open access publishing is a thriving and sustainable aspect of STM publishing. The number of subscription-based journals transferring to BioMed Central's open access publishing platform increased in 2008, further substantiating the move toward open access publishing. *Osteopathic Medicine and Primary Care *is well positioned to benefit from the combined experiences of BioMed Central and Springer in open access publishing.

Articles in *Osteopathic Medicine and Primary Care *are universally accessible online without charge. Thus, clinicians and researchers anywhere may view our journal website to access current and archived publications. In 2009, *Osteopathic Medicine and Primary Care *remains the only major osteopathic journal to provide universal open access to all content, all the time [10]. The short lag time in bringing articles to press is another advantage of our open access publishing. The median time from initial manuscript submission to publication of peer-reviewed articles in 2008 was four months (126 days). Our goal in 2009 will be to further reduce this lag time to three months (90 days).

The National Institutes of Health (NIH) announced and implemented their revised policy on enhancing public access to archived publications resulting from NIH-funded research in 2008 [11]. The associated law states: "*The Director of the National Institutes of Health shall require that all investigators funded by the NIH submit or have submitted for them to the National Library of Medicine's PubMed Central an electronic version of their final, peer-reviewed manuscripts upon acceptance for publication, to be made publicly available no later than 12 months after the official date of publication: Provided, That the NIH shall implement the public access policy in a manner consistent with copyright law*." Publishing in *Osteopathic Medicine and Primary Care *is a simple and efficient way for NIH grantees to ensure full compliance with the relevant policies.

## Article processing charges and waivers

*Osteopathic Medicine and Primary Care *levies article processing charges (APCs), after a manuscript submission has been peer-reviewed and accepted for publication, to cover the cost of operations (rejected manuscripts are not subject to APCs). Authors at institutions that are members of BioMed Central will receive either a full waiver or partial discount of the APCs depending on their institutional membership. *Osteopathic Medicine and Primary Care *also maintains a journal fund to support waivers of APCs for authors who are not affiliated with BioMed Central member institutions and who do not otherwise have access to grant funds or local monies to support the cost of publication. All waivers, regardless of funding mechanism, must be requested at the time of initial manuscript submission. The two primary criteria in determining whether a waiver is awarded are the quality of the submitted work and its congruence with our scope of interest.

## Peer review and reader comments

*Osteopathic Medicine and Primary Care *strives to provide quick and fair review of all manuscripts. We are indebted to the 24 reviewers who completed evaluations of one or more manuscripts during 2008 (Appendix). We encourage readers of *Osteopathic Medicine and Primary Care *to post comments on our articles via the hyperlinks provided on the title page or last page of the full text version of articles (reader comment hyperlinks are not available in the PDF version of articles). Comments relating to clinical practice generally will not be posted without supporting evidence from peer-reviewed publications. Figures, tables, and equations cannot be included within a posted comment; however, hyperlinks to such elements may be included. Upon acceptance all reader comments are directly linked to the full text version of the relevant article. Reader comments are not indexed in PubMed or archived in PubMed Central. General comments or correspondence relating to *Osteopathic Medicine and Primary Care *may be sent to the Editor-in-Chief at OMPC@hsc.unt.edu.

## Book reviews

In response to author and publisher demands, *Osteopathic Medicine and Primary Care *will begin posting book reviews in the "Latest news" section of its home page in 2009. Books within our scope of interest are most likely to be reviewed. The book reviews will not be indexed in PubMed or archived in PubMed Central. Please submit books for review to: John C. Licciardone, Editor-in-Chief, *Osteopathic Medicine and Primary Care*, University of North Texas Health Science Center, 3500 Camp Bowie Boulevard, Fort Worth, TX 76107, USA. There is no guarantee that a received book will be reviewed, and *Osteopathic Medicine and Primary Care *will be unable to return any book, reviewed or unreviewed. Manuscript submissions will not be accepted at this address. In addition to book reviews, we expect to roll out other regular recurring features in the "Latest news" section of our home page during 2009.

## Conclusion

*Osteopathic Medicine and Primary Care *continues to promote and advance open access publishing in 2009 through universal online access without charge, indexing in PubMed and archiving in PubMed Central, retention of authors' copyright, and expeditious peer review. We encourage manuscript submissions and reader comments via our journal website.

## Competing interests

The author declares that he has no competing interests.

## Appendix

*Osteopathic Medicine and Primary Care *peer reviewers during 2008: Margaret Aguwa, Denise Burns, Roberto Cardarelli, Nancy Cooper, Gautam Desai, Christian Fossum, Gary Fryer, Kimberly Fulda, Frederick Goldstein, Kendi Hensel, Hollis King, Margot Kurtz, John Licciardone, Nicholas Lucas, Ron Marino, Reza Nassiri, Raymond Perrin, Karl-Ludwig Resch, Paula Scariati, Michael Seffinger, Peggy Smith-Barbaro, Karen Steele, Richard Virgilio, Nefyn Williams.
